# Assessment of Age-Related Morphometric Changes of Subcortical Structures in Healthy People Using Ultra-High Field 7 Tesla Magnetic Resonance Imaging

**DOI:** 10.3389/fnagi.2016.00224

**Published:** 2016-09-26

**Authors:** Xue-Yuan Wang, Lei Zhao, Tao Yu, Liang Qiao, Duan-Yu Ni, Guo-Jun Zhang, Yong-Jie Li

**Affiliations:** ^1^Beijing Institute of Functional Neurosurgery, Xuanwu Hospital, Capital Medical UniversityBeijing, China; ^2^Department of Neurology, Huanhu HospitalTianjin, China

**Keywords:** aging, thalamus, third ventricle, ultra-high field, magnetic resonance imaging, mammillothalamic tract

## Abstract

**Objective**: To assess the age-related morphometric changes of subcortical structures in healthy people.

**Materials and Methods**: Ultra-high field 7 tesla magnetic resonance (MR) imaging in humans was used to visualize the subcortical structures of healthy young, middle-aged and elderly participants. Using the magnetization-prepared two rapid acquisition gradient echo (MP2RAGE) sequence, we assessed the visibility of the margins of the thalamus and white matter in the thalamus, as well as the anterior commissure (AC) and posterior commissure (PC) length, the maximal height of the thalamus, the half width of the third ventricle and the distance between the AC and the center of the mammillothalamic tract (MTT) at the level of the AC-PC plane. All quantitative data were statistically evaluated.

**Results**: The AC-PC length did not differ significantly among the three groups. The maximal height of the thalamus decreased with age (*r_s_*_(53)_ = −0.719, *p* < 0.001). The half width of the third ventricle (*r_s(53)_* = 0.705, *p* < 0.001) and the distance between the AC and the center of the MTT (*r_s(53)_* = 0.485, *p* < 0.001) increased with age. The distance between the AC and the center of the MTT of the young and the elderly participants differed significantly (*p* = 0.007).

**Conclusion**: The AC-PC length is not a good candidate for proportional correction during atlas-to-patient registration. The maximal height of the thalamus and the half width of the third ventricle correlated strongly with age, and the MTT position in relation to the AC shifted posteriorly as age increased. These age-related morphometric changes of subcortical structures should be considered in targeting for functional neurosurgery.

## Introduction

Deep brain stimulation (DBS) is an attractive alternative therapy for patients with various movement disorders, including advanced Parkinson’s disease and essential tremor, and other drug-resistant neurological diseases, including epilepsy and Tourette’s syndrome (Wichmann and DeLong, 2016). Previously, when there was a lack of quality brain imaging, atlases of the human brain were used to guide stereotaxic procedures, including the Schaltenbrand-Wahren (Schaltenbrand and Wahren, 1977) and the Schaltenbrand-Bailey (Schaltenbrand and Bailey, 1959) atlases, the Talairach and Tournoux atlas (Talairach and Tournoux, 1988), the Morel atlas (Morel, 2007) and the Mai atlas (Mai et al., 2015).

Despite the development of remarkable neuroradiological technologies, these atlases have continued to play a critical role in DBS surgery, especially since the targets of interest are not yet typically displayed by the clinical magnetic resonance (MR) scanner currently used. Nonetheless, it has been found that the same stereotactic coordinates did not necessarily designate the same anatomical loci in these atlases (Niemann et al., 2000), even in the different microscopic series of the same atlas (Niemann et al., 1994; Niemann and van Nieuwenhofen, 1999). It is unclear which factors cause such inconsistency—different degrees of tissue shrinkage, inter-individual neuroanatomical variability, or age-related morphometric changes. First, the authors of these atlases claimed that the freezing method used for histological preparation resulted in a shrinkage of only approximately one percent (Schaltenbrand and Wahren, 1977), and a shrinkage factor was evaluated and corrected with the help of the corresponding MR images (Morel, 2007). Therefore, this factor can be excluded. However, since most available atlases are the result of anatomic studies made on a limited number of cadaver brains of different ages, the dimensional variation caused by aging cannot be excluded. For instance, the Mai atlas (Mai et al., 2015) was derived from the brain of a 24-year-old male, the SW atlas (Schaltenbrand and Wahren, 1977) was based on the brains of a 40-year-old male (LXXVIII) and a 57-year-old male (LXVIII), the Morel atlas (Morel et al., 1997; Morel, 2007) was derived from a 59-year-old female brain (Hb7) and two 70-year-old female brains (Hb1 and Hb2), and the Talairach and Tournoux atlas (Talairach and Tournoux, 1988) was based on a 60-year-old female brain. More importantly, the thalamic height in the young brain used for the Mai atlas (22 mm) was larger than that in the middle-aged brains (Hb7, LXXVIII, and LXVIII) and in the brain used for the Talairach and Tournoux atlas (a range of 19 to 20.5 mm). Furthermore, the thalamic height was similar in the elderly brains (Hb1 and Hb2; a range of 15 to 15.5 mm). According to such a sparse number of post-mortem studies, we hypothesized that there was a tendency for the thalamic height to decrease with aging.

Therefore, we evaluated the morphologic information about the subcortical structures by means of ultra-high field 7 tesla (T) MR imaging in healthy people of different ages. We felt that such a study might be helpful for understanding the problems encountered with the registration of stereotaxic atlases to patient MR images.

## Materials and Methods

All datasets used in this section were downloaded from Dryad (Forstmann et al., 2014), and all are publicly available datasets stripped of identifiers that do not require Institutional Review Board review referred to the Ethics Committee of Xuanwu Hospital, Capital Medical University. The protocol of this study has been approved by the Ethics Committee of Xuanwu Hospital, Capital Medical University.

### Participants

The 7 T dataset consisted of 53 usable subjects: 30 young subjects (15 females) with a mean age of 24 (standard deviation (SD): 2.3), 12 middle-aged subjects (six females) with a mean age of 51 (SD: 6.2), and 11 elderly subjects (four females) with a mean age of 68 (SD: 5.6) were included (Table 1). All participants had normal or corrected-to-normal vision, and none of them suffered from neurological, psychiatric, or somatic diseases. All subjects were right-handed, as confirmed by the Edinburgh Inventory.

**Table 1:** **Mean measurements in millimeters for the three age groups**.

	*N*	Age (minimum-maximum) (years)	AC-PC length (mm)	Maximal thalamic height (mm)	Half width of the third ventricle (mm)	MTT-AC distance (mm)
Young	30	24 ± 2.3 (19–28)	26.0 ± 1.02	19.6 ± 0.66	2.1 ± 0.41	11.5 ± 0.59
Middle-aged	12	51 ± 6.2 (40–59)	26.0 ± 1.14	18.1 ± 0.71	2.7 ± 0.65	11.9 ± 0.54
Elderly	11	68 ± 5.6 (60–75)	25.8 ± 0.77	17.0 ± 1.24	4.0 ± 0.63	12.2 ± 0.74
F^a^ value			0.175	42.373	53.644	5.639
*P*^a^			0.840	<0.001	<0.001	0.006
*P*^b^ (Young vs. Elderly)				<0.001	<0.001	0.007
*P*^b^ (Middle-aged vs. Elderly)				<0.001	<0.001	0.531
*P*^b^ (Young vs. Middle-aged)				<0.001	<0.001	0.136

*AC, anterior commissure; PC, posterior commissure; MTT, mammillothalamic tract; ^a^One-way ANOVA. ^b^Tukey post hoc test*.

### MRI Scan Parameters

Structural data were acquired using a 7 T Siemens Magnetom MRI scanner and a 24-channel head array Nova coil (NOVA Medical Inc., Wilmington, MA, USA), and a whole-brain magnetization-prepared two rapid acquisition gradient echo (MP2RAGE) sequence was analyzed. The whole-brain MP2RAGE had 240 sagittal slices with an acquisition time (TA) of 10:57 min (repetition time (TR) = 5000 ms; echo time (TE) = 2.45 ms; inversion times (TI1/TI2) = 900/2750 ms; flip angle = 5°/3°; bandwidth = 250 Hz/Px; voxel size = (0.7 mm)^3^). The INV1 volume reflects the gradient echo sequence with an TI of 900 ms. For the high contrast between the gray matter and the white matter with the INV1 volume, all our measurements were conducted with this sequence.

### Preprocess of MRI

**Figure 1:**
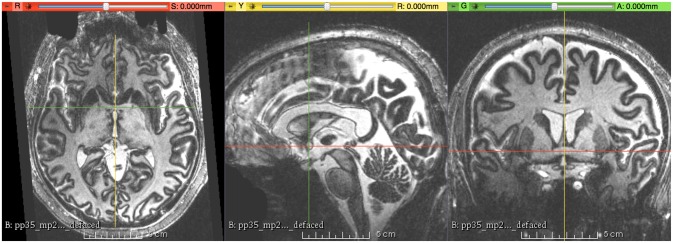
**This figure shows the final result of magnetic resonance (MR) images aligned to the right-anterior-superior (RAS) coordinate system, with the origin (*R* = 0, *A* = 0, *S* = 0) at the center of the anterior commissure (AC)**.

MR image alignment to the Right-Anterior-Superior (RAS) coordinate system was carried out with the software package 3D-Slicer (Version 4.5^1^) using the Transforms module. The first step is to load the MRI volume into Slicer. The contrast values in the viewer for the image were then set to increase the visibility of the structural borders between the gray matter and the white matter. The MR images were then rotated along the three axes (left-right, anterior-posterior, and superior-inferior) to make the midsagittal plane strictly vertical and the anterior commissure (AC)-posterior commissure (PC) line parallel to the anterior-posterior axis. Finally, when necessary, the MR images were subjected to translation to move the center of the AC to the origin of the RAS coordinate system using the transformation matrix that was generated by the Fiducial Registration module in Slicer; consequently, the following corresponding measurements were directly read from the coordinates of the cursor (Figure 1). In a coarse-to-fine strategy (steps of 0.5° were used), the alignment was finally performed at 10× magnification under visual control. Alignment of the MRI volume was subjectively determined based on the consensus of two raters (XW and LZ). After that, the file names of the participants were randomly renamed by another author (DN) to mask the ages of these participants.

### Measurements

**Figure 2:**
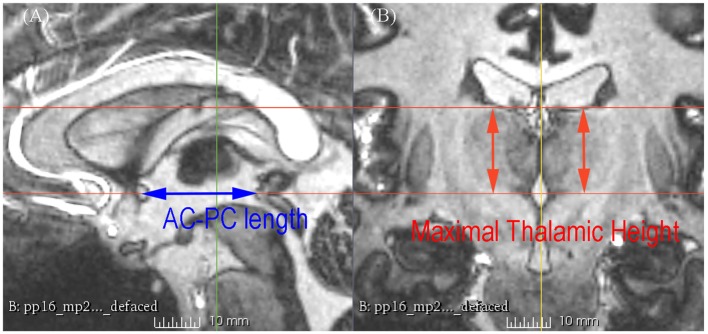
**7 T MRI showing the measurement of the AC-posterior commissure (PC) length and the maximal thalamic height on the sagittal section (A) and the coronal section (B)**.

**Figure 3:**
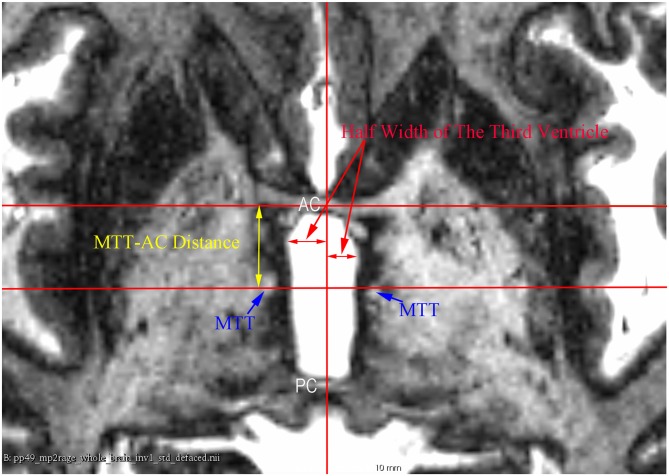
**An axial 7 T MR image showing the measurement of the half width of the third ventricle and the mammillothalamic tract (MTT)-AC distance at the AC-PC plane**.

The subcortical structures were measured by two raters (XW and LZ) as follows (Figures 2, 3): all coronal sections were scrutinized from anterior to posterior, and the maximal height of the thalamus was recorded as the maximal S coordinate of the cursor at the upper limit of the thalamus. Interactive refinement of the measurement was supported by a simultaneous visualization of three orthogonal sections and the position of the cursor in these three sections. The half width of the third ventricle was measured at the AC-PC plane, and the maximum width was chosen regardless of its relative distance to the AC or PC; most of the time, it was just posterior to the AC or anterior to the PC, and corresponding values of the left side and right side were averaged for convenience. The mammillothalamic tract (MTT)-AC distance was measured as the distance between the center of the MTT and the center of AC at the AC-PC plane; corresponding values of the left side and right side were also averaged for convenience. An inter-rater reliability test of the AC-PC length, the maximal height of the thalamus, the half width of the third ventricle, and the distance between the AC and the center of the MTT was carried out using the intra-class correlation coefficient (ICC), which were 0.982 (95% CI 0.945–0.994; *p* < 0.001), 0.914 (95% CI 0.731–0.972; *p* < 0.001), 0.986 (95% CI 0.957–0.996; *p* < 0.001), and 0.944 (95% CI 0.826–0.982; *p* < 0.001), respectively. Because of the high inter-rater reliability, the results of two raters were averaged.

### Statistical Analysis

All statistical analyses were performed using the Statistical Package for the Social Sciences (SPSS; SPSS Inc., Chicago, IL, USA) version 17.0 for Windows. The statistical significance threshold was set at *P* < 0.05. Group differences were analyzed using analysis of variance (ANOVA) and corrected for multiple comparison using Tukey HSD. A series of Spearman’s rank-order correlations were conducted on the whole subjects to determine if there were any relationship among the AC-PC length, the maximal height of the thalamus, the half width of the third ventricle, the MTT-AC distance, and the age and sex of the subjects. Independent samples *t*-tests were used to evaluate differences in measurements between male and female subjects.

## Results

### Qualification

**Figure 4:**
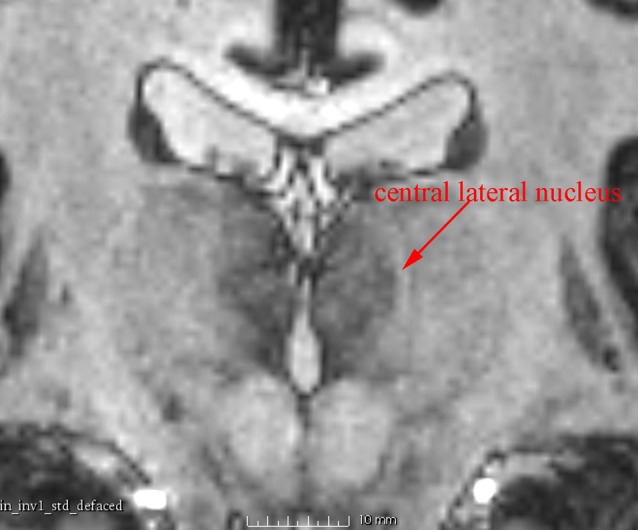
**A coronal 7 T MR image showing the central lateral nucleus, a structure about 1 mm wide, which is clearly identified as a high-intensity zone**.

In addition to the enhanced contrast, 7 T MRI has the potential to illustrate the thin inter-nuclear borders that separate individual nuclei in the thalamus (Figure 4). This was very useful to differentiate adjacent nuclei with otherwise close signal.

### Age-Related Differences

There was no significant difference among groups in terms of AC-PC length as determined by one-way ANOVA (*F*_(2,50)_ = 0.175, *p* = 0.840) as shown in Table 1. However, the three groups differed significantly in terms of maximal thalamic height (*F*_(2,50)_ = 42.373, *p* < 0.001), the half width of the third ventricle (*F*_(2,50)_ = 53.644, *p* < 0.001), and the MTT-AC distance (*F*_(2,50)_ = 5.639, *p* = 0.006). A Tukey *post hoc* test revealed that the MTT-AC distance was shorter in the young group (11.5 ± 0.59 mm, *p* = 0.007) than in the elderly group (12.2 ± 0.74 mm). However, no statistically significant differences in MTT-AC distance were found between the young and middle-aged groups (11.9 ± 0.54 mm, *p* = 0.136) or between the middle-aged and the elderly groups (*p* = 0.531). A Tukey *post hoc* test revealed that the maximal thalamic height was higher in the young (19.6 ± 0.66 mm, *p* < 0.001) and middle-aged groups (18.1 ± 0.71 mm, *p* < 0.001) than in the elderly group (17.0 ± 1.24 mm). There was also a statistically significant difference between the young and middle-aged groups (*p* < 0.001). A Tukey *post hoc* test revealed that the half width of the third ventricle was wider in the young (2.1 ± 0.41 mm, *p* < 0.001) and middle-aged groups (2.7 ± 0.65 mm, *p* < 0.001) than in the elderly group (4.0 ± 0.63 mm). There was also a statistically significant difference between the young and middle-aged groups (*p* < 0.001).

**Figure 5:**
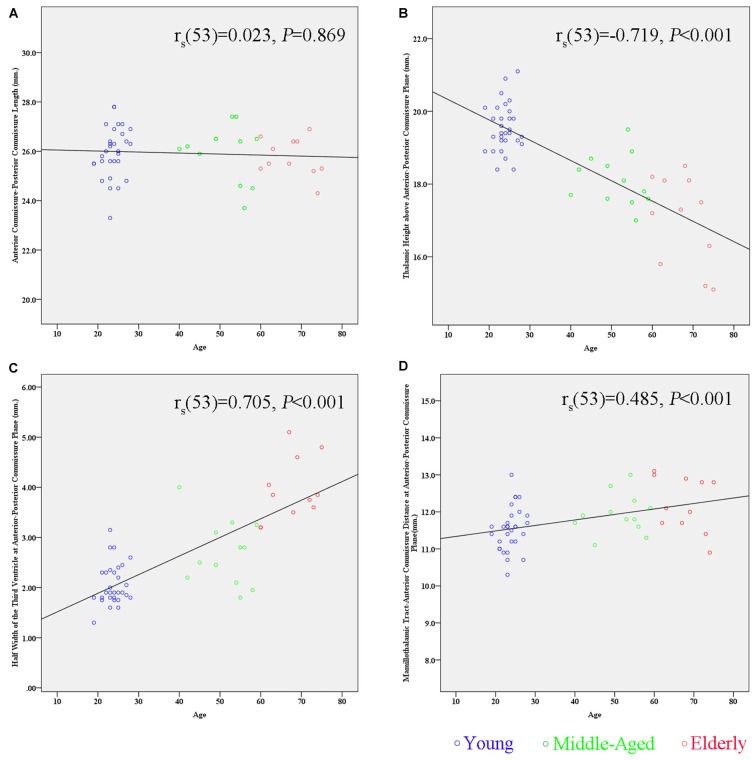
**Scatter plots showing the relationship between age and the measurement of subcortical structures. (A)** There is no significant correlation between the AC-PC length and age (*r_s_*_(53)_ = 0.023, *p* = 0.869). **(B)** There is a significant negative correlation between the maximal thalamic height and age (*r_s(53)_* = −0.719, *p* < 0.001). **(C)** There is a significant positive correlation between the half width of the third ventricle and age (*r_s(53)_* = 0.705, *p* < 0.001). **(D)** There is a significant positive correlation between the MTT-AC distance and age (*r*_s(53)_ = 0.485, *p* < 0.001).

By correlating these measurements separately with age, we found that the maximal thalamic height, the half width of the third ventricle, and the MTT-AC distance correlated highly with age (the maximal thalamic height: *r_s_*_(53)_ = −0.719, *p* < 0.001; the half width of the third ventricle: *r_s(53)_* = 0.705, *p* < 0.001; the MTT-AC distance: *r_s(53)_* = 0.485, *p* < 0.001; Table 2). The correlations revealed shrinkage of the height of the thalamus, an expansion of the third ventricle, and a posterior shift of MTT position with increasing age (Figure 5).

**Table 2:** **Correlations between the measurements obtained from 7 T MR images**.

	AC-PC length	Maximal thalamic height	Half width of the third ventricle	MTT-AC distance
Age	0.023	−0.719**	0.705**	0.485**
Sex	−0.532**	−0.005	−0.192	−0.330*
AC-PC length		0.157	0.031	0.541**
Maximal thalamic height			−0.658**	−0.187
Half width of the third ventricle				0.391**

AC, anterior commissure; PC, posterior commissure; MTT, mammillothalamic tract; **P < 0.01. *P < 0.05.

### Gender Differences

**Figure 6:**
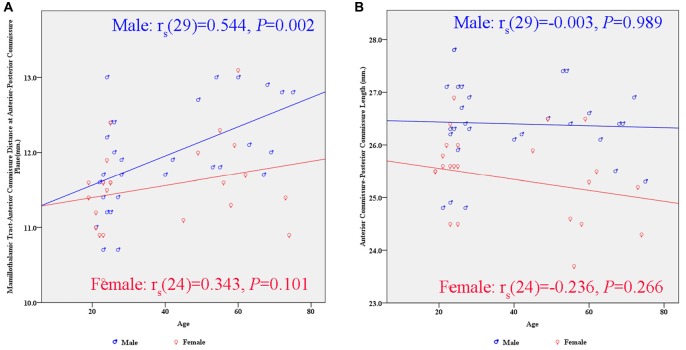
**Scatter plots showing the gender differences in the relationship between subcortical structures and age. (A)** There is a significant positive correlation between the MTT-AC distance and age in the male group (*r*_s__(29)_ = 0.544, *p* = 0.002). **(B)** There is no significant correlation between the AC-PC length and age in either gender.

There were significant differences between males and females in terms of AC-PC length and MTT-AC distance (Table 3). In each case, mean values for males were greater than for females (Figure 6). There were no significant differences between males and females in terms of maximal thalamic height and the half width of the third ventricle.

**Table 3:** **Gender differences in the mean values of anatomical measurements**.

	Male		Female		*P*^a^
	Mean	(SD)	Mean	(SD)
*N*	29		24		N/A
Age (years)	40	(18.9)	38.0	(19.30)	NS
AC-PC Length	26.4	(0.80)	25.4	(0.90)	<0.001
Thalamic height	18.8	(1.20)	18.6	(1.46)	NS
Half width of the third ventricle	2.8	(1.00)	2.4	(0.74)	NS
MTT-AC distance	12.0	(0.68)	11.5	(0.59)	<0.05

*Standard deviation values are given in parentheses, and all measurements are in millimeters. AC, anterior commissure; PC, posterior commissure; MTT, mammillothalamic tract; NS, not significant. ^a^ Independent samples t test*.

## Discussion

Rapid developments in MR scanners and software have made MRI a tool of increasing value in the study of the effects of healthy aging on the brain. In the present study, young, middle-aged and elderly subjects were structurally scanned using ultra-high resolution 7 T MR. Age-related volume reduction of the thalamus has been detected in recent years using *in vivo* MRI of healthy participants with ages varying across the adult age span (Raz and Rodrigue, 2006; Fjell and Walhovd, 2010). Although the mechanisms of age-related shrinkage are unclear, it is important to recognize factors that may affect the target position in order to improve the efficacy and safety of stereotactic surgery.

In a post-mortem study, den Dunnen and Staal (2005) measured the length of the AC-PC line in the brains of 12 patients who had died of non-neurological diseases. They found that the length of the AC-PC line was almost constant (mean: 24.4 mm; SD: 3.58 mm) during life, in accordance with the findings of the present study (mean: 25.9 mm; SD: 0.98 mm). A proportional factor of correction according to the length of the AC-PC line has been suggested for the stereotactic atlas registration (Nowinski, 2004). Our finding of a lack of correlation between AC-PC distance and age is in agreement with the findings of Van Buren (Van Buren and Maccubbin, 1962); thus, scaling of the atlas to this distance seems to result in mismatch along the other two perpendicular axes (Niemann et al., 2000).

Our finding of a positive correlation between the half width of the third ventricle and age is in agreement with the findings of Good et al. (2001) and Kitajima et al. (2008). Walhovd et al. (2005) also reported significant age-related differences in all neuroanatomical volumes, including the thalamus and the third ventricle. However, the volume change is not equal to width change. Frequently, the width of the third ventricle is not the same anteriorly as posteriorly (Dormont et al., 1997). We hypothesize that the boundary between the stable AC-PC and the unstable subcortical gray structure may be the most sensitive area influenced by age. Our results also confirmed this hypothesis. Dilatation of the third ventricle associated with increasing age in normal subjects has the potential to change the target position of DBS procedures. subthalamic nucleus (STN) position has been reported to move laterally during aging. For instance, Pereira et al. (2016) evaluated STN position in 134 patients with Parkinson’s disease (ages 28–84), finding that the third ventricle width and age are statistically significant predictors of radiographic STN lateralization. On the other hand, using the same dataset as ours, Keuken et al. (2013) showed that age, but not third ventricle volume, is significantly correlated with the lateral location of the STN.

Significant differences in the MTT-AC distance were only found between young and elderly participants. With increasing age, the center of MTT tends to move posteriorly. A recent human study on the long-term benefit of anterior nucleus DBS for epilepsy demonstrated that the efficacious target for the control of seizure frequency is localized in close proximity to the MTT (Krishna et al., 2016). Because the lower part of the MTT is located laterally to the third ventricle and the upper part of the MTT is located underneath the anterior nucleus, its posterior shift and the age-related changes in the width of the third ventricle and the height of the thalamus should all be taken into account during targeting of the anterior nucleus in DBS procedures for epilepsy.

The present results indicate that the height of the thalamus was strongly negatively associated with age. Most of the previous studies are limited to the volume change of the thalamus. Only piecemeal information was available for the relationship of the height of the thalamus with age. In a previous post-mortem study (Van Buren and Maccubbin, 1962), Van Buren performed autopsies on 16 patients (ages 3–78) without neurological diseases and showed that the thalamic height above the AC-PC line was in a range of 16.5 to 21 mm. The thalamus is a highly differentiated gray matter structure, and reduction of its height can obviously change the position of its subnuclei, especially in the dorsal-ventral direction. Our finding of no sex differences in the age-related reduction of the height of the thalamus is consistent with a recent study using volumetric measurement (Van Der Werf et al., 2001). The age-related change of the height of the thalamus has been overlooked in previous studies. For example, Niemann et al. (2000) reported the generation of an internally consistent Canonical Model of Morel’s atlas normalized with the sagittal model. During atlas-to-patient registration, even when completing the proportional correction along the AC-PC line, the atlas had to be scaled by 1.25 in the dorsal-ventral direction for better matching with the thalamic surface contours. The displacement of subcortical structures associated with aging is more like a shift of the axes of the structures in three dimensional space, which necessitates the creation of an age-specific stereotactic atlas, as Fillmore et al. (2015) performed for MR studies. The scarcity of age-appropriate atlases for functional neurosurgery cannot be resolved at this time (Sadikot et al., 2011); however, fortunately, intraoperative microelectrode recording and test stimulation can be used for guidance to some targets of DBS.

There are some limitations to this study that should be kept in mind when interpreting our results. The first limitation involves the restricted number of subjects; due to the unavailability of an ultra-high field MR scanner for clinical investigation in China, only the publicly available dataset was used. It has been reported that including a wider age range may change the linear correlation to a curvilinear one, but young children or people older than 80 are usually not candidates for DBS procedures. In addition, our study is a cross-sectional study. Longitudinal studies are capable of identifying subtle age-related morphometric changes during aging by controlling for individual differences. To our knowledge, no longitudinal study has yet looked at the age-related changes of these subcortical structures. Therefore, further longitudinal studies will be especially important.

## Conclusion

These findings are of crucial importance to neuroscientists and neurosurgeons. Mapping of human electrophysiological data onto a stereotactic atlas based solely on the AC-PC length for proportional spatial normalization without stratifying factors—such as the width of the third ventricle and age—has potential hazards, especially concerning small structures. We suggest that these age-related morphometric changes should also be taken into account when localizing the subcortical target during clinical DBS procedures.

## Author Contributions

Y-JL, TY, LQ, D-YN, G-JZ and X-YW designed and conducted the study, including data collection and data analysis. LZ, TY, LQ, D-YN, G-JZ and X-YW conducted the data collection and data analysis. X-YW and LZ prepared the manuscript draft with important intellectual input from Y-JL. All authors approved the final manuscript. Y-JL, LZ and X-YW had complete access to the study data.

## Conflict of Interest Statement

The authors declare that the research was conducted in the absence of any commercial or financial relationships that could be construed as a potential conflict of interest.

## Footnotes

^1^http://www.slicer.org/

## References

[B1] den Dunnen W. F. Staal M. J. (2005). Anatomical alterations of the subthalamic nucleus in relation to age: a postmortem study. Mov. Disord. 20, 893–898. 10.1002/mds.20417 15809991

[B2] Dormont D. Cornu P. Pidoux B. Bonnet A. M. Biondi A. Oppenheim C.. (1997). Chronic thalamic stimulation with three-dimensional MR stereotactic guidance. Am. J. Neuroradiol. 18, 1093–1107. 9194437 PMC8337308

[B3] Fillmore P. T. Phillips-Meek M. C. Richards J. E. (2015). Age-specific MRI brain and head templates for healthy adults from 20 through 89 years of age. Front. Aging Neurosci. 7:44. 10.3389/fnagi.2015.00044 25904864 PMC4389545

[B4] Fjell A. M. Walhovd K. B. (2010). Structural brain changes in aging: courses, causes and cognitive consequences. Rev. Neurosci. 21, 187–221. 10.1515/revneuro.2010.21.3.187 20879692

[B5] Forstmann B. U. Keuken M. C. Schafer A. S. Bazin P. Alkemade A. Turner R. (2014). Multi-modal ultra-high resolution structural 7-Tesla MRI data repository. Sci. Data 1:140050. 10.1038/sdata.2014.50 [Dryad Digital Repository. 10.5061/dryad.fb41s] 25977801 PMC4421933

[B6] Good C. D. Johnsrude I. S. Ashburner J. Henson R. N. Friston K. J. Frackowiak R. S. (2001). A voxel-based morphometric study of ageing in 465 normal adult human brains. Neuroimage 14, 21–36. 10.1006/nimg.2001.0786 11525331

[B7] Keuken M. C. Bazin P. L. Schäfer A. Neumann J. Turner R. Forstmann B. U. (2013). Ultra-high 7T MRI of structural age-related changes of the subthalamic nucleus. J. Neurosci. 33, 4896–4900. 10.1523/jneurosci.3241-12.2013 23486960 PMC6619019

[B8] Kitajima M. Korogi Y. Kakeda S. Moriya J. Ohnari N. Sato T.. (2008). Human subthalamic nucleus: evaluation with high-resolution MR imaging at 3.0 T. Neuroradiology 50, 675–681. 10.1007/s00234-008-0388-4 18443775

[B9] Krishna V. King N. K. Sammartino F. Strauss I. Andrade D. M. Wennberg R. M.. (2016). Anterior nucleus deep brain stimulation for refractory epilepsy: insights into patterns of seizure control and efficacious target. Neurosurgery 78, 802–811. 10.1227/neu.0000000000001197 26813858

[B10] Mai J. K. Majtanik M. Paxinos G. (2015). Atlas of the Human Brain. San Diego, CA: Academic Press.

[B11] Morel A. (2007). Stereotactic Atlas of the Human Thalamus and Basal Ganglia. New York, NY: Informa Healthcare USA, Inc.

[B12] Morel A. Magnin M. Jeanmonod D. (1997). Multiarchitectonic and stereotactic atlas of the human thalamus. J. Comp. Neurol. 387, 588–630. 10.1002/(SICI)1096-9861(19971103)387:4<588::AID-CNE8>3.0.CO;2-Z 9373015

[B14] Niemann K. Mennicken V. R. Jeanmonod D. Morel A. (2000). The Morel stereotactic atlas of the human thalamus: atlas-to-MR registration of internally consistent canonical model. Neuroimage 12, 601–616. 10.1006/nimg.2000.0650 11112393

[B15] Niemann K. Naujokat C. Pohl G. Wollner C. von Keyserlingk D. (1994). Verification of the Schaltenbrand Wahren stereotactic atlas. Acta Neurochir. (Wien) 129, 72–81. 10.1007/bf01400876 7998500

[B13] Niemann K. van Nieuwenhofen I. (1999). One atlas—three anatomies: relationships of the Schaltenbrand Wahren microscopic data. Acta Neurochir. (Wien) 141, 1025–1038. 10.1007/s007010050479 10550646

[B16] Nowinski W. L. (2004). Co-registration of the Schaltenbrand-Wahren microseries with the probabilistic functional atlas. Stereotact. Funct. Neurosurg. 82, 142–146. 10.1159/000081346 15467381

[B17] Pereira J. L. B A S. Sharim J. Yazdi D. DeSalles A. A. Pouratian N. (2016). Lateralization of the subthalamic nucleus with age in Parkinson’s disease. Basal Ganglia 6, 83–88. 10.1016/j.baga.2016.01.003 26900546 PMC4755314

[B18] Raz N. Rodrigue K. M. (2006). Differential aging of the brain: patterns, cognitive correlates and modifiers. Neurosci. Biobehav. Rev. 30, 730–748. 10.1016/j.neubiorev.2006.07.001 16919333 PMC6601348

[B19] Sadikot A. F. Chakravarty M. M. Bertrand G. Rymar V. V. Al-Subaie F. Collins D. L. (2011). Creation of computerized 3D MRI-integrated atlases of the human basal ganglia and thalamus. Front. Syst. Neurosci. 5:71. 10.3389/fnsys.2011.00071 21922002 PMC3167101

[B20] Schaltenbrand G. Bailey P. (1959). Introdution to Stereotaxis with an Atlas of the Human Brain. Stuttgart: Georg Thieme.

[B21] Schaltenbrand G. Wahren W. (1977). Atlas for Stereotaxy of the Human Brain. Stuttgart: Georg-Thieme-Verlag.

[B22] Talairach J. Tournoux P. (1988). Co-planar Stereotaxic Atlas of the Human Brain. 3-Dimensional Proportional System: An Approach to Cerebral Imaging. New York, NY: Georg Thieme Verlag.

[B23] Van Buren J. M. Maccubbin D. A. (1962). An outline atlas of the human basal ganglia with estimation of anatomical variants. J. Neurosurg. 19, 811–839. 10.3171/jns.1962.19.10.0811 13995970

[B24] Van Der Werf Y. D. Tisserand D. J. Visser P. J. Hofman P. A. Vuurman E. Uylings H. B.. (2001). Thalamic volume predicts performance on tests of cognitive speed and decreases in healthy aging. A magnetic resonance imaging-based volumetric analysis. Cogn. Brain Res. 11, 377–385. 10.1016/s0926-6410(01)00010-6 11339987

[B25] Walhovd K. B. Fjell A. M. Reinvang I. Lundervold A. Dale A. M. Eilertsen D. E.. (2005). Effects of age on volumes of cortex, white matter and subcortical structures. Neurobiol. Aging 26, 1261–1270; discussion 1275–1268. 10.1016/j.neurobiolaging.2005.05.020 16005549

[B26] Wichmann T. DeLong M. R. (2016). Deep brain stimulation for movement disorders of basal ganglia origin: restoring function or functionality? Neurotherapeutics 13, 264–283. 10.1007/s13311-016-0426-6 26956115 PMC4824026

